# Multivitamin use may lower risk of preeclampsia: A meta‐analysis

**DOI:** 10.1111/aogs.14403

**Published:** 2022-06-09

**Authors:** Andre C. Q. Lo, Charmaine Chu Wen Lo

**Affiliations:** ^1^ University of Cambridge School of Clinical Medicine, Addenbrooke's Hospital Cambridge UK; ^2^ Liverpool Hospital Liverpool New South Wales Australia; ^3^ University of New South Wales Faculty of Medicine Kensington New South Wales Australia; ^4^ Western Sydney University School of Medicine Penrith New South Wales Australia

Sir,

There is increasing interest in the use of vitamins and minerals for preeclampsia prophylaxis. On current evidence, however, the systematic review by Christiansen et al.^1^ was unable to draw “any final conclusions…regarding a preventive effect of multivitamin use in relation to preeclampsia”.

Nevertheless, we believe the existing literature indicates that multivitamins may reduce the risk of preeclampsia. To start with, considering the limited evidence base identified by Christiansen et al.,^1^ we see no reason to exclude studies investigating foods fortified with multiple vitamins/minerals, provided the control group is given placebo food without fortification.^2^, ^3^ We also believe that Chen et al.,^4^ despite being excluded for having “wrong outcomes”, can be included since their “severe PIH” outcome is consistent with the diagnostic criteria for preeclampsia with severe features.^5^ Lastly, we are not convinced that it is inappropriate to pool results from trials with differences in population or timing of intervention, though this may require exploration with stratification/sensitivity analyses and/or downgrading of GRADE based on indirectness.

Accordingly, we performed a random effects meta‐analysis including the aforementioned randomized controlled trials (RCTs).^2^, ^3^, ^4^ The statistics of Christiansen et al.^1^ (REVMAN) were replicated using the meta package in R (Version 4.0.5) and the DerSimonian–Laird estimator for between‐study variance. Our meta‐analysis of RCTs indicate that multivitamins reduce the risk of preeclampsia, though most trials were conducted in women with increased risk of the condition (Figure 1). Sensitivity analyses including only women with increased risk of preeclampsia or excluding studies investigating fortified foods remained significant and reported lowered risk estimates.

**FIGURE 1:**
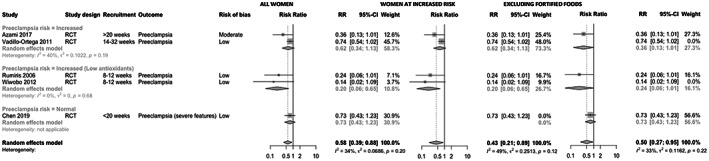
Meta‐analysis of interventional data examining the effect of multivitamin use on risk of preeclampsia irrespective of baseline preeclampsia risk (left), examining only women with increased risk of preeclampsia (middle), and excluding studies with supplementation from fortified foods (right), with stratification for risk of preeclampsia.

Turning to the non‐significant meta‐analysis of adjusted observational data by Christiansen et al.,^1^ we believe Catov et al. can be included, as HR/RRs approximate ORs under the rare disease assumption. Hence, periconceptional/first trimester multivitamin use is associated with significantly reduced preeclampsia incidence (Figure 2). Although there was evidence of moderate between‐study heterogeneity, this is likely explained in part by different study methodologies. For example, although Catov et al. and Høgh et al. both examined Danish populations, the former excluded single supplement and irregular multivitamin use, whereas the latter's control group included any individual who answered “no” to daily multivitamin use, which could explain the latter's elevated odds ratios. A sensitivity analysis including Chen et al.^4^ in this meta‐analysis (since they also examined a non‐high‐risk population) did not materially change the risk estimate but reduced heterogeneity (RR = 0.80, 95% CI = 0.67–0.95; *I* ^2^ = 39%, *P* = 0.13). Meanwhile, subgroup analyses found a reduced risk of preeclampsia with multivitamin use in women with a body mass index (BMI) ≥25 but not in women with BMI <25.

**FIGURE 2:**
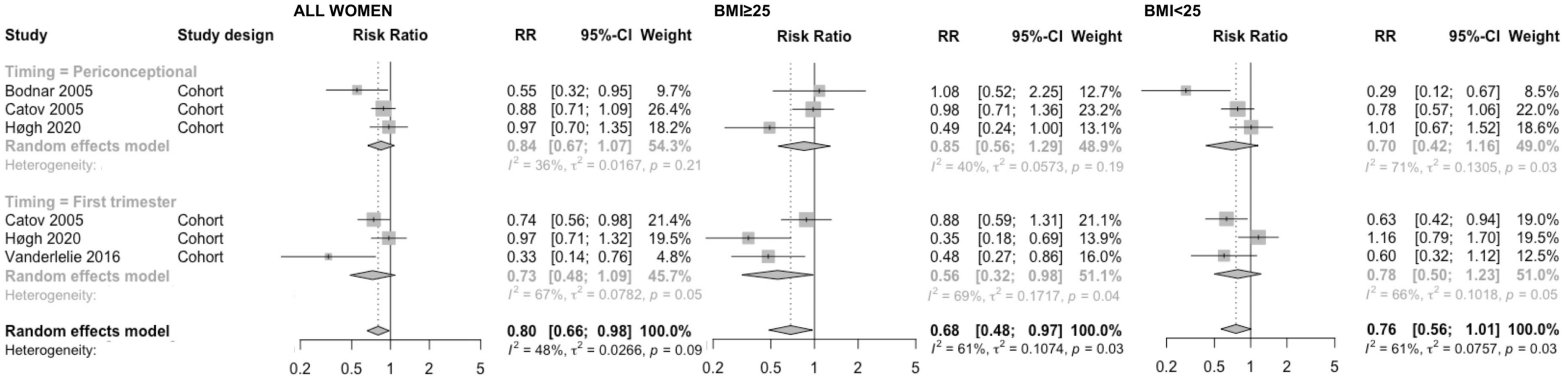
Meta‐analysis of adjusted observational data examining the effect of multivitamin use on risk of preeclampsia for all women (left), for women with BMI ≥25 (middle), and for women with BMI <25 (right), with stratification for timing of multivitamin use.

In conclusion, there is evidence of moderate certainty that multivitamin use may be beneficial for women at increased risk of preeclampsia (including having low antioxidant levels), with downgrading of evidence due to differences in population and intervention. However, the certainty of evidence for multivitamin use in healthy women is low, being based largely on observational data. Nevertheless, it is unclear whether the decreased risk is driven by certain multivitamin components (eg calcium^6^) and what vitamin and mineral combinations may confer the most benefit (eg vitamin C and E combinations may not be effective^7^). Further clarification of these aspects is warranted.

## AUTHOR CONTRIBUTIONS

ACQL and CCWL conceived the study, and wrote and reviewed the manuscript. ACQL performed the statistical analyses.
